# Divorce and risk of suicide attempt: a Swedish national study

**DOI:** 10.1017/S0033291723003513

**Published:** 2023-12-12

**Authors:** Alexis C. Edwards, Henrik Ohlsson, Jessica E. Salvatore, Mallory E. Stephenson, Casey Crump, Jan Sundquist, Kristina Sundquist, Kenneth S. Kendler

**Affiliations:** 1 Department of Psychiatry, Virginia Institute for Psychiatric and Behavioral Genetics, Virginia Commonwealth University School of Medicine, Richmond, VA, USA;; 2 Center for Primary Health Care Research, Lund University, Malmö, Sweden; 3 Department of Psychiatry, Robert Wood Johnson Medical School, Rutgers University, Piscataway, NJ, USA; 4 Department of Family Medicine and Community Health, Icahn School of Medicine at Mount Sinai, New York, NY, USA; 5 Department of Population Health Science and Policy, Icahn School of Medicine at Mount Sinai, New York, NY, USA

**Keywords:** divorce, familial confounding, suicide attempt, survival model

## Abstract

**Background.:**

Prior research has reported an association between divorce and suicide attempt. We aimed to clarify this complex relationship, considering sex differences, temporal factors, and underlying etiologic pathways.

**Methods.:**

We used Swedish longitudinal national registry data for a cohort born 1960–1990 that was registered as married between 1978 and 2018 (*N* = 1 601 075). We used Cox proportional hazards models to estimate the association between divorce and suicide attempt. To assess whether observed associations were attributable to familial confounders or potentially causal in nature, we conducted co-relative analyses.

**Results.:**

In the overall sample and in sex-stratified analyses, divorce was associated with increased risk of suicide attempt (adjusted hazard ratios [HRs] 1.66–1.77). Risk was highest in the year immediately following divorce (HRs 2.20–2.91) and declined thereafter, but remained elevated 5 or more years later (HRs 1.41–1.51). Divorcees from shorter marriages were at higher risk for suicide attempt than those from longer marriages (HRs 3.33–3.40 and 1.20–1.36, respectively). In general, HRs were higher for divorced females than for divorced males. Co-relative analyses suggested that familial confounders and a causal pathway contribute to the observed associations.

**Conclusions.:**

The association between divorce and risk of suicide attempt is complex, varying as a function of sex and time-related variables. Given evidence that the observed association is due in part to a causal pathway from divorce to suicide attempt, intervention or prevention efforts, such as behavioral therapy, could be most effective early in the divorce process, and in particular among females and those whose marriages were of short duration.

## Introduction

Across both historical and contemporary theories of suicidal thoughts and behaviors, a lack of social integration or connectedness features prominently as a risk factor. Durkheim (1897) wrote of ‘egoistic suicide’ as that which arose from a lack of integration in one’s community; ‘thwarted belongingness’ is a necessary but insufficient component of suicide risk in the interpersonal theory of suicide (Joiner, 2005; Van Orden et al., 2010); and the three-step theory highlights a lack of connectedness as a key element in the transition from suicidal ideation to action (Klonsky & May, 2015). Thus, divorce – which signals the dissolution of an important, legally recognized, and frequently long-term romantic relationship – has the potential to precipitate suicidality. Consistent with this view, previous research supports an increased risk of suicide attempt (SA) as a function of divorce to varying degrees, with odds ratios (ORs) ranging from 1.56 in Puerto Rico to 7.14 in West Germany (Weissman et al., 1999), and with risks potentially higher within East Asian *v.* European cultures (Yip, Yousuf, Chan, Yung, & Wu, 2015). Furthermore, meta-analytic data indicate that risk of suicide death is higher among divorced individuals compared to those who are married (OR 4.09, 95% confidence interval [CI] 3.97–4.22) (Kyung-Sook, SangSoo, Sangjin, & Young-Jeon, 2018).

Despite divorce being a well-documented correlate of suicidal behavior, the nature of this association is not yet clearly understood. Clinically relevant questions remain, including the degree to which the divorce–suicide association is likely to be due to causal effects or confounding factors, such as social class and comorbid internalizing (e.g. major depression and anxiety disorder) and externalizing (e.g. alcohol and drug use disorders and criminal behavior) disorders (Kessler, Borges, & Walters, 1999; Kessler, Walters, & Forthofer, 1998; Thomas et al., 2022; Whisman, Salinger, & Sbarra, 2022). Likewise, genetic confounding may contribute to the association. Both suicidal behavior and divorce are, in part, genetically influenced, with heritability estimates ranging from 0.17 to 0.55 (Brent & Melhem, 2008; Edwards et al., 2021; Fu et al., 2002; Voracek & Loibl, 2007) and 0.13 to 0.52 (McGue & Lykken, 1992; Salvatore et al., 2017; Salvatore, Larsson Lönn, Sundquist, Sundquist, & Kendler, 2018), respectively. Importantly, there is prior evidence that divorce is genetically correlated with other forms of psychopathology including alcohol use disorder (Salvatore et al., 2017), major depression (Kendler & Karkowski-Shuman, 1997), and a personality composite characterized by behavioral disinhibition (Jocklin, McGue, & Lykken, 1996). Disentangling causal *v.* shared liability pathways is necessary to determine the most effective targeted prevention/intervention efforts.

Alongside the need to clarify the nature of the association between divorce and suicidal behavior is the need to better understand who may be at elevated suicide risk following divorce and how to appropriately time interventions. Men typically benefit more from the salutary health effects of marriage than women (Kiecolt-Glaser & Newton, 2001; Kiecolt-Glaser & Wilson, 2017; Umberson, 1992), and accordingly the loss of marriage through divorce may be expected to have a more detrimental impact on men. Consistent with these findings, the point estimate for the divorce–suicide association was stronger in males (OR 3.80, 95% CI 1.98–7.31) compared to females (1.77, 95% CI 0.82–3.82) in a recent meta-analysis (Kyung-Sook et al., 2018), though the CIs overlapped. Genetic factors may render some individuals particularly susceptible to the pathogenic effects of divorce. Contextual triggering and diathesis-stress perspectives (Shanahan & Hofer, 2005), which posit that disease/disorder is the result of contextual stressors combined with an underlying predisposition, suggest that those at genetic risk for suicidal behavior may be especially susceptible to SA following divorce. Finally, divorce is a process rather than a discrete event (Amato, 2010) and developing preventive interventions to reduce suicidal behavior among divorcing individuals necessitates a careful understanding of the underlying temporal dynamics. There is some evidence that the year following divorce represents an especially high-risk period (Jamison, Bol, & Mintz, 2019), and that separation is even more strongly associated with suicide death than divorce (Wyder, Ward, & De Leo, 2009).

In the current study, we asked five questions probing the nature of the association between divorce and SA using nation-wide Swedish registry data, which afford a representative record of divorce and potentially important covariates and confounders.

1. At the population level, what is the association between divorce and SA, and is the association equal across the sexes?
2. Is the divorce–SA association robust to behavioral and genetic confounders?
3. Is the divorce–SA association stronger among those with higher (*v.* lower) genetic predispositions to SA?
4. Does the divorce–SA association depend on marital duration or time since divorce?
5. Using a co-relative model design, which compares exposures and outcomes within families while controlling genetic and shared environmental factors shared by relatives, can we determine the degree to which the divorce–SA association is likely causal?

## Materials and methods

### Sample

We collected longitudinal information on individuals from Swedish population-based registers with national coverage linking each person’s unique personal identification number which, to preserve confidentiality, was replaced with a serial number by Statistics Sweden. We secured ethical approval for this study from the Regional Ethical Review Board in Lund (No. 2008/409 and later amendments). In the database, we included all individuals born in Sweden between 1960 and 1990 who were registered as married sometime between 1978 and 2018.

### Measures

SA was defined in the Swedish medical registers. We focus on non-fatal SA, rather than SA and death, due to prior evidence of outcome-specific etiologies (Edwards et al., 2021). We used the first date of SA registration. SA registrations that were followed by a registration in the mortality register within one week were not considered as SA to avoid misclassifying as non-fatal attempts that ultimately resulted in death. In the database, we also included date of divorce, mean parental education, age at marriage, date of birth of first child, and registrations of externalizing and internalizing behavior. We further included a family genetic risk score for SA (FGRS_SA_), which has been described previously (Edwards et al., 2023; Kendler, Ohlsson, Sundquist, & Sundquist, 2021a, 2021b). Details on FGRS derivation, together with definitions of all covariates, are provided in the online Supplementary Material.

### Statistical analyses

#### Cox proportional hazards model

In the primary analyses, we used Cox proportional hazards models to investigate the association between divorce and SA. We report the hazard ratio (HR) and 95% CIs. In these models, we follow individuals from date of marriage until end of the follow-up (SA, death, emigration, or 12–31-2018, whatever came first). We treated divorce as a time-dependent covariate (i.e. until the date of the divorce the individual was considered free of exposure, while from the date of divorce the individual was considered exposed until end of follow-up). In model A, we included divorce, year of birth, sex at birth, parental education, age at marriage, and the child variable (which was treated as a time-dependent covariate). In model B, we further included information on externalizing and internalizing registrations as time-dependent covariates. In model C, we added FGRS_SA_, and in model C2, we interacted FGRS_SA_ with divorce.

Alongside this multiplicative interaction, we present the additive interaction, using relative excess risk due to interaction (RERI) (Richardson & Kaufman, 2009) and the synergy index (SI) (Rothman, Greenland, & Lash, 2008), to provide insight into whether new cases of SA will be produced when individuals are exposed to both divorce and high FGRS beyond what would be expected from the impact of the two factors on their own. This is best represented by an additive interaction (Kendler & Gardner, 2010). Additional details on Cox models are provided in the online Supplementary Material. All analyses were performed using SAS 9.4 (©2002–2012 SAS Institute Inc., Cary NC, USA).

#### Co-relative models

Using a co-relative design (Kendler, Ohlsson, Sundquist, & Sundquist, 2014), we examined if the regression results (i.e. the association between divorce and SA) reflected confounding by familial risk factors. From the Swedish Multi-Generation and Twin Registers, we identified all monozygotic (MZ) twin, full- and half-sibling, and cousin pairs. Using stratified Cox proportional hazards models, with a separate stratum for each relative pair, we refitted the analysis to adjust for a range of unmeasured genetic and environmental factors shared within the relative pair as described previously (Edwards, Ohlsson, Sundquist, Sundquist, & Kendler, 2020; Kendler, Lonn, Salvatore, Sundquist, & Sundquist, 2017; Kendler et al., 2014). Additional details are provided in the online Supplementary Material.

#### Difference-in-difference model

To assess SA rates across time for cases and controls, we used a difference-in-difference model as part of a series of exploratory analyses. From the database, we selected all individuals that were registered for a divorce (i.e. cases) and matched them to three controls who were not divorced at the time of the case’s divorce, based on the following variables: year of birth, sex, child, age at marriage (±1 year), and FGRS_SA_ quartiles based on k-means clustering. The divorce had to occur prior to 2012–12-31, allowing for least 6 years of follow up. We then examined SA rates across time for cases and controls. Additional details are provided in the online Supplementary Material.

## Results

### Descriptive statistics and preliminary analyses

The cohort of married individuals born in Sweden between 1960 and 1990 consisted of *N* = 1 601 075, among whom *N* = 412 002 (25.7%) divorced during the observation period. Further details are provided in Table 1. The prevalence of first SA was higher among divorced individuals. These individuals were also older, had married at a younger age, had lower mean parental education levels, were more often parents, were more likely to have registrations at baseline for externalizing and internalizing disorders, and had higher FGRS_SA_.

### Association between divorce and suicide attempt

We conducted a series of Cox regression models to estimate the association between divorce and SA, as shown in Table 2. In model A, for the full sample, we first estimated the effect of divorce while controlling for potentially important sociodemographic covariates and observed a robust association (HR = 2.94; 95% CI 2.85–3.02). Because model A indicated that males were significantly more likely to attempt suicide, in model A2, we tested an interaction term between divorce and sex. Divorced males were less likely to attempt suicide than divorced females (interaction HR = 0.83 [0.79–0.87]); we therefore provide findings for the sexes combined (including sex as a covariate) and also stratified by sex to facilitate interpretation.

We next tested whether the observed associations could be accounted for by potential behavioral or biological confounders. In model B, we controlled for externalizing and internalizing registrations, which attenuated the effect size of divorce considerably. In model C, we added FGRS_SA_ as a covariate and observed a significant association with SA, though this addition did not measurably impact the magnitude of the association between divorce and SA. Finally, in model C2, we included an interaction term between divorce and FGRS_SA_. The estimate was significantly lower than 1 on the multiplicative scale; however, when converting to measures of an additive interaction – the RERI and SI – we did not observe a significant interaction.

The results from sex-stratified models conducted pursuant to model A2 above are provided in Table 2. In model A, the association between divorce and SA was higher in females (HR = 3.17; 95% CI 3.05–3.30) than in males (HR = 2.66; 95% CI 2.55–2.77). Including externalizing and internalizing registrations as covariates led to reduced HRs for divorce in model B. In model C, further correcting for FGRS_SA_ had little effect on the HR for divorce. As in the combined-sex analysis, we observed deviations from multiplicativity of the divorce-by-FGRS_SA_ term, but no significant deviations from additivity for either sex.

### Length of marriage and time since divorce

**Figure 1.**
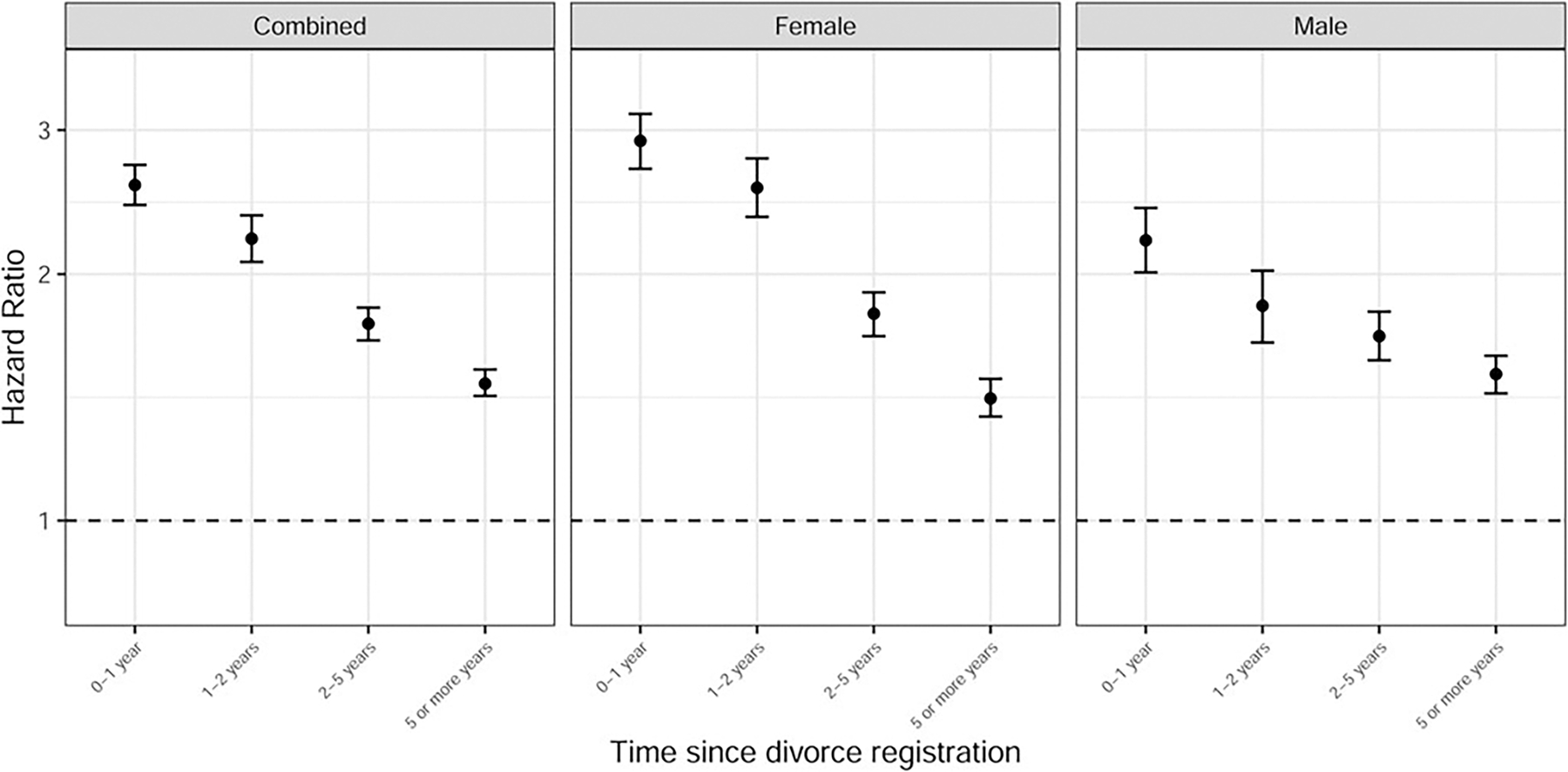
Associations between divorce and suicide attempt as a function of time since divorce registration. Hazard ratios and 95% confidence intervals are presented for four non-overlapping time frames since date of divorce registration: up to 1, 1–2, 2–5, and 5 more years. Estimates are adjusted for sociodemographic covariates and registrations for externalizing and internalizing (i.e. model C). Complete results are available in online Supplementary Table S3. *Y*-axis is on the log scale. Black dashed horizontal line at HR = 1 represents the null hypothesis.

We next estimated the association between divorce and SA across different time frames subsequent to divorce registration. These models were adapted from model C, which adjusted for sociodemographic covariates along with externalizing and internalizing registrations. As shown in Fig. 1 and online Supplementary Table S3, HRs declined as more time elapsed.

We also evaluated whether the association between divorce and SA varied as a function of the length of marriage, estimating HRs for five marriage-length bins as described in the Supplementary Methods section. In models of the sexes combined and for sex-stratified analyses, HRs were highest for individuals with the shortest marriages (up to 2.7 years in length; HRs 3.33–3.40) and lowest for those with the longest marriages (22.6 years or longer; HRs 1.20–1.36). In all cases, HRs remained significantly above 1 (see online Supplementary Table S4 for complete results).

### Co-relative analyses

**Figure 2.**
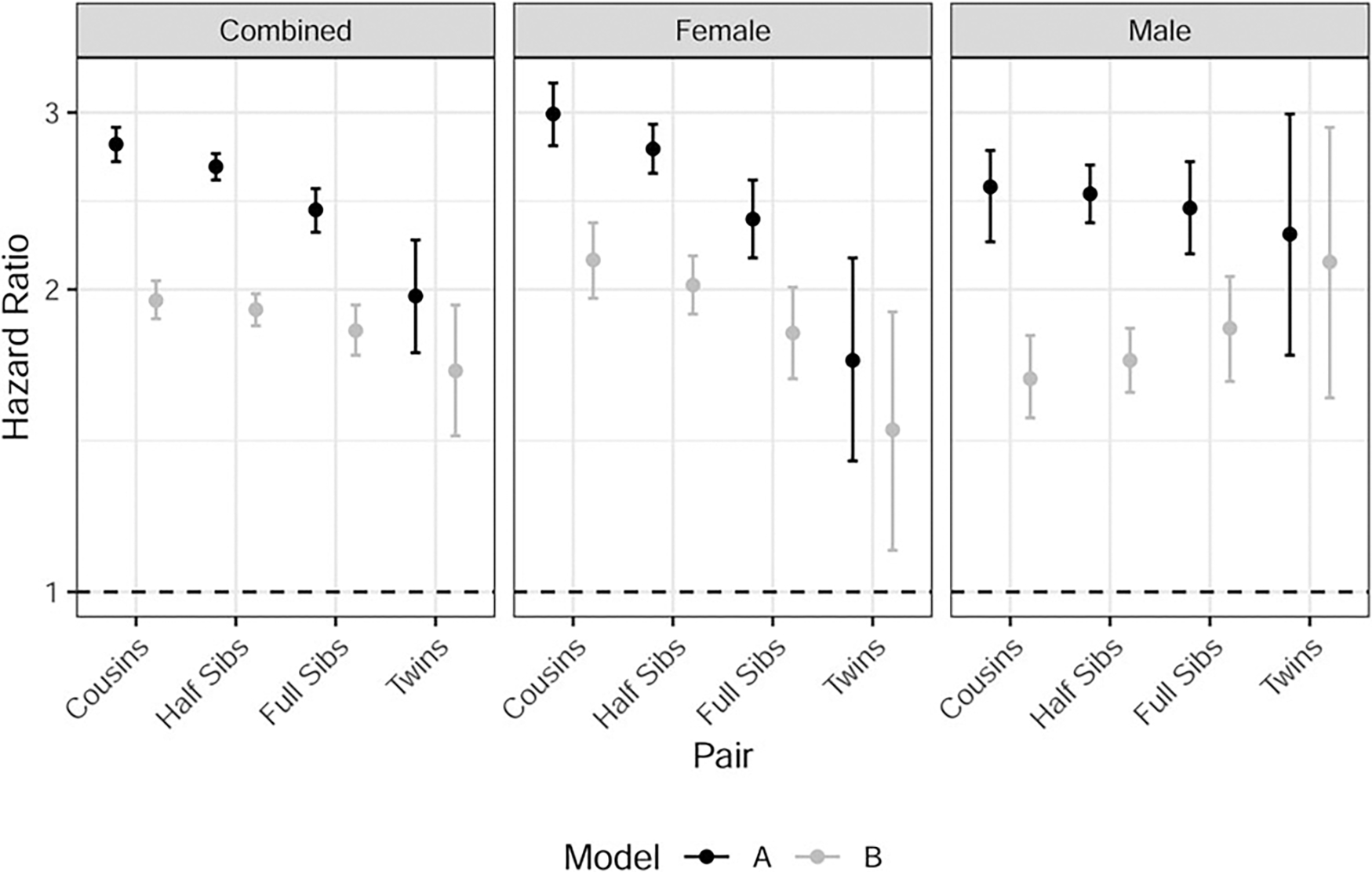
Co-relative model results. Hazard ratios and 95% confidence intervals are presented for the predicted estimates of each relative pair. Results are presented for the sexes combined and for sex-stratified analyses. Estimates from model A are adjusted for sex at birth (combined panel only), year of birth, parental education^1^, parenthood status, and age at marriage. Estimates from model B are additionally adjusted for externalizing and internalizing registrations. *Y*-axis is on the log scale. Black dashed horizontal line at HR = 1 represents the null hypothesis. ^1^As these models only compare individuals within the same strata, and parental education does not vary for full siblings or monozygotic twins, it is only relevant for cousins and half-sibling pairs. Furthermore, year of birth and sex are not relevant covariates for monozygotic twins.

To evaluate the extent to which the association between divorce and SA was attributable to a causal pathway *v.* confounding familial factors that jointly increase risk for both, we specified co-relative models. Complete results are provided in online Supplementary Table S5. Akaike’s information criterion values were superior in the predicted model in all but one case (online Supplementary Table S5). Results from the predicted models are depicted in Fig. 2, for both model A (adjusted for sociodemographic covariates) and model B (further adjusted for externalizing and internalizing registrations). In model A, the HRs declined modestly with increasing degrees of genetic relatedness, ranging from 1.70 to 2.27 in MZ twin pairs across samples (sexes combined, females, and males). Estimates were slightly attenuated with the inclusion of externalizing and internalizing covariates (model B) but remained above 1 in each case. The decline across pairs of higher relatedness was more pronounced among females than males, suggesting that familial confounding factors contribute more to the association between divorce and SA among females. However, as HRs remained above 1 in twins, these data are consistent with a residual causal pathway contributing to the association. Note that observed data on twin pairs were sparse, leading to imprecise HR estimates and CIs that in some cases spanned 1 (online Supplementary Table S5).

### Exploratory analyses

We conducted a difference-in-difference test, wherein we examined the rate of SA across time. As shown in the online Supplementary Figure, actual SA rates among individuals who divorced during the observation period departed from the expected rates beginning 1–2 years prior to the divorce registration date, peaked in the year prior to registration, and declined thereafter. Based on these findings, we conducted a series of exploratory analyses wherein we considered the onset of ‘exposure’ to begin 2 years prior to formal divorce registration. Results are provided in the online Supplementary Material text and online Supplementary Tables S6–S9; HRs between divorce and attempt were modestly increased in these analyses but otherwise we observed no substantive differences. We further investigated the possibility that an SA might precipitate divorce (i.e. the direction of effect could be inverted from our original tests). These analyses are described in the online Supplementary Material text and online Supplementary Tables S10–S11. SA was associated with divorce (HR = 1.54 in adjusted models), with evidence of causality in co-relative models. Finally, we tested whether adjusting for spousal psychopathology and SA impacted the overall effect of divorce in model C. These models, which included spousal registrations as time-dependent covariates, are reported in online Supplementary Table S12. While the main effects of spousal psychopathology on proband SA were positive, their inclusion resulted in only slight attenuations to the effect of divorce.

## Discussion

In this study of a large birth cohort of married Swedish individuals, we sought to provide context for previous observations that risk of SA is elevated among divorced individuals. Our series of analyses yielded findings that clarify the magnitude of risk in a representative cohort and have important implications for our understanding of the timing of, and etiologic pathways underlying, the association between divorce and SA. First, we observed a robust positive association between divorce and SA, and this effect was more pronounced among females. Second, the observed effect was attenuated but remained significant even after accounting for comorbid psychopathology and genetic liability. Third, in primary analyses, risk of SA was not exacerbated in individuals at higher genetic liability to SA (i.e. there was no deviation from additivity). Fourth, the risk for attempt declined as time elapsed since divorce registration, though it persisted for at least 5 years; furthermore, individuals with shorter marriages were at higher risk. Finally, familial confounding factors contribute to the association between divorce and SA, but did not fully account for it, supporting a potentially causal pathway. These findings underscore the complexity of the potentially adverse effects of divorce and provide empirical support for the centrality of interpersonal connection in historical and contemporary theories of suicidality.

Even after adjusting for sociodemographic factors and psychopathology, divorced individuals were nearly twice as likely to attempt suicide (HRs = 1.70–1.83) as their married counterparts. In comparison, HRs for psychopathology were 3.22–5.68, underscoring the centrality of psychiatric illness in risk for suicidal behavior (though many suicide decedents do not have a known history of psychiatric disorders [Stone et al., 2018]). In contrast with prior studies of divorce and suicide death (Kyung-Sook et al., 2018), females fared more poorly after divorce than males. This could be due to disproportionate losses in household income, increased risk of poverty, and greater likelihood of single parenting in women *v.* men following divorce (Leopold, 2018).

Our analysis on the duration of risk indicates that, perhaps unsurprisingly, the period immediately following divorce is likely an important target for prevention/intervention efforts, particularly for females. However, the HRs remained elevated even 5 or more years after divorce, demonstrating that the upheaval of divorce is related to persistent negative outcomes; furthermore, exploratory analyses indicate increased SA risk in the period immediately preceding divorce, though estimation of when marital discord begins is not feasible using registry data. Information regarding correlates of marriage length in well-powered studies is sparse, precluding clear hypotheses around our observation that divorcées from shorter marriages were at higher risk for SA. However, the first 7 years of marriage are widely regarded as volatile (Gottman & Levenson, 2002). Although speculative, this volatility may translate into extreme behaviors, such as suicidality, in the wake of divorce, whereas ending a longer-term troubled marriage could be less problematic. We again observed different patterns across the sexes: females with shorter marriages were at higher risk of SA, while those with longer marriages were at lower risk, relative to their male peers. These findings speak to the complexity of sex differences in the context of stressful events, such as divorce, and psychopathology. Prior studies have suggested that males are more susceptible to depression and suicidality after divorce (Evans, Scourfield, & Moore, 2016; Kendler, Thornton, & Prescott, 2001; Kolves, Ide, & De Leo, 2010; Kposowa, 2003). The discordance with the current findings could be due to our ability to control for a wide range of covariates, differences across countries/cultural contexts, or other factors, and should be further dissected in future studies.

The divorce–SA association could be attributable to confounding genetic factors and/or familial environmental exposures that are associated with both outcomes, such as childhood abuse/neglect (Brown, Cohen, Johnson, & Smailes, 1999; Colman & Widom, 2004; Zatti et al., 2017). Our co-relative analyses demonstrate that familial confounding factors do play a role: as we accounted for greater genetic similarity and environmental sharing in related pairs, HRs declined. Importantly, point estimates were consistently >1 even in our most conservative models, and CIs spanned 1 only where our statistical power was lowest (using observed data in sex-stratified analyses). These results are consistent with a modest causal effect of divorce on risk of SA.

Interestingly, in the co-relative model using predicted estimates, familial confounding played a more prominent role in accounting for the divorce–SA association for females than for males: in model A, the HRs declined from 2.99 to 1.70 for females, and from 2.53 to 2.27 for males. In model B, which accounted for comorbid psychopathology, we again observed greater attenuation of HRs among females who were increasingly related; among males, HRs actually increased slightly. Ultimately, though the overall analyses indicate that divorce has a stronger impact on risk of attempt among females, the *causal* component of risk is stronger among males.

Although divorce is, overall, associated with higher risk of SA, it is not monolithic in terms of its sequelae. A review of divorce and health outcomes notes that the *modal* effect of divorce is psychosocial resilience (Sbarra, 2015). Indeed, one study found that >70% of participants exhibited stably high levels of subjective well-being after divorce, while another 9% reported increases in well-being (Mancini, Bonanno, & Clark, 2011). Other research has found that individuals who initiate a divorce are likely to adapt better afterwards (Hewitt & Turrell, 2011); that those with higher education or better financial conditions were more likely to be resilient after divorce (Perrig-Chiello, Hutchison, & Morselli, 2014); and that divorced women were more likely than their unhappily married female peers to be professionally successful and have high levels of self-worth and self-efficacy (Hetherington, 2003). Thus, positive outcomes post-divorce are not uncommon. In contrast, specific subgroups of individuals not examined could be especially susceptible to the negative impact of divorce or other stressful life events. For example, prior studies have found that individuals with alcohol use disorder are at high risk for suicide in the context of stressors (Conner et al., 2012; Murphy, Wetzel, Robins, & McEvoy, 1992), including loss of an interpersonal relationship (Murphy, Armstrong, Hermele, Fischer, & Clendenin, 1979). Such nuances warrant direct testing in future research.

Our findings should be interpreted in the context of several limitations. First, we do not have data on when marital problems began or when/if couples separated prior to divorce (parents who seek to divorce must undergo a waiting period; see online Supplementary Table S1). We attempted to capture this in our exploratory analyses by considering the period prior to formal divorce as the onset of exposure, but this is necessarily an imperfect approach. If marital discord rather than only divorce *per se* contributes to risk, this could lead to underestimates of the effect size, as many couples will experience discord but remain married, and therefore be classified as controls. Future work would benefit from prospective studies that include self-reports of marital discord, including among couples that remain married, which could enable disentangling of the effects of discord *v.* divorce.

Second, while co-relative models account for confounding genetic and familial environmental factors, they do not correct for non-familial exposures that could jointly increase risk of divorce and SA. Thus, the MZ-based HRs from these models should be considered the upper bound of a potential causal effect of divorce. The current finding of a causal pathway could potentially be validated through the use of other methods that enable causal inference, for example, Mendelian randomization. We note, however, the challenge of identifying valid instrumental variables in the context of highly polygenic outcomes such as suicidal behavior and divorce.

Third, our findings are specific to the cohort we selected to maximize data availability and follow-up time, and might not generalize to individuals in other cohorts or countries, particularly given cultural differences and shifting societal norms surrounding divorce. Similarly, we compared only married and divorced individuals: additional analyses will be necessary to assess risk of SA among those who are widowed or never married. Though outside the scope of the current study, national registry data in Sweden and elsewhere can be used in this manner in future studies; such studies can also be expanded to examine the magnitude of effect of divorce on risk of suicide death.

In conclusion, our findings are consistent with prior research indicating that divorce is associated with increased risk of suicidal behavior. We substantively build on that work by demonstrating that this association is attributable to both a causal pathway and to confounding familial factors; the causal pathway appears more prominent among males. In contrast with findings from some other studies on the consequences of divorce, females fared worse after divorce than their male peers. However, risk approached baseline more quickly after divorce for females. Overall, these findings – including our difference-in-difference model – suggest that screening for marital discord, or whether a couple is contemplating divorce, could be an important step toward identifying those at risk of suicidal behavior; furthermore, suicide prevention resources might be most fruitfully targeted at those whose divorces are relatively recent, particularly among individuals with other risk indicators such as psychopathology or a short duration marriage. While divorce can present an opportunity for positive change, this frequently stressful event can lead to serious negative outcomes including suicidal behavior.

## Supplementary Material

Supplementary Material

## Figures and Tables

**Table 1.** Descriptive statistics of analytic cohort, consisting of married individuals who were born in Sweden 1960–1990

	Full sample		Females		Males	
	Not divorced	Divorced	Not divorced	Divorced	Not divorced	Divorced
*N*	1 189 073	412 002 (25.7%)	611 116	225 879 (27.0%)	577 957	186 123 (24.4%)
Suicide attempt	9.778 (0.8%)	16 522 (4.0%)	4656 (0.8%)	9511 (4.2%)	5122 (0.9%)	7.011 (3.8%)
Mean year of birth (s.d.)	1973 (6.5)	1969 (6.9)	1973 (8.3)	1969 (7.1)	1973 (7.9)	1969 (6.6)
Female	51.4%	54.8%	n/a	n/a	n/a	n/a
Mean age at marriage (s.d.)	31.9 (6.6)	28.3 (5.6)	30.9 (6.5)	27.2 (5.3)	33.0 (6.5)	29.7 (5.6)
Mean parental education (s.d.)	11.4 (2.9)	10.7 (2.7)	11.4 (2.9)	10.7 (2.7)	11.4 (2.9)	10.7 (2.7)
Has a child	90.9%	91.5%	91.8%	92.2%	89.9%	90.6%
Externalizing registration	10.8%	23.5%	5.9%	15.7%	15.9%	32.7%
Internalizing registration	19.3%	34.0%	25.3%	41.7%	13.0%	24.7%
Mean FGRS_SA_^a^ (s.e.)	−0.06 (0.08)	0.09 (1.0)	−0.05 (0.8)	0.10 (1.0)	−0.06 (0.8)	0.06 (0.9)

SD, standard deviation; FGRS_SA_, family genetic risk score for suicide attempt.

a FGRS_SA_ is standardized by birth year and county of residence.

**Table 2.** Hazard ratios and 95% confidence intervals from Cox regressions estimating the association between divorce and first suicide attempt

Full sample	Model A	Model A2	Model B	Model C	Model C2
Divorce	2.94 (2.85; 3.02)	3.19 (3.08; 3.31)	1.78 (1.73; 1.84)	1.73 (1.68; 1.79)	1.80 (1.74; 1.85)
Year of birth	1.03 (1.02; 1.03)	1.03 (1.02; 1.03)	0.99 (0.99; 0.99)	0.99 (0.99; 0.99)	0.99 (0.99; 0.99)
Sex (M *v*. F)	1.11 (1.08; 1.13)	1.19 (1.16; 1.23)	0.98 (0.95; 1.00)	0.98 (0.96; 1.01)	0.98 (0.96; 1.01)
Parental education	0.94 (0.93; 0.94)	0.94 (0.93; 0.94)	0.96 (0.96; 0.97)	0.97 (0.96; 0.97)	0.97 (0.96; 0.97)
Child	0.86 (0.83; 0.89)	0.86 (0.83; 0.89)	0.86 (0.83; 0.89)	0.84 (0.81; 0.87)	0.84 (0.81; 0.87)
Age at marriage	0.99 (0.99; 0.99)	0.99 (0.99; 0.99)	0.96 (0.96; 0.96)	0.96 (0.96; 0.97)	0.96 (0.96; 0.96)
Sex×divorce		0.83 (0.79; 0.87)			
Externalizing			4.16 (4.05; 4.28)	3.93 (3.82; 4.04)	3.93 (3.82; 4.04)
Internalizing			4.92 (4.77; 5.08)	4.77 (4.62; 4.99)	4.77 (4.62; 4.92)
FGRS_SA_				1.23 (1.22; 1.24)	1.28 (1.27; 1.29)
FGRS_SA_×divorce					0.92 (0.90; 0.93)^a^
Females	Model A	Model A2	Model B	Model C	Model C2
Divorce	3.17 (3.05; 3.30)	n/a	1.83 (1.75; 1.90)	1.77 (1.69; 1.84)	1.85 (1.77; 1.93)
Year of birth	1.02 (1.02; 1.02)	n/a	0.97 (0.97; 0.97)	0.97 (0.97; 0.97)	0.97 (0.97; 0.97)
Parental education	0.95 (0.94; 0.95)	n/a	0.97 (0.96; 0.97)	0.97 (0.97; 0.98)	0.97 (0.97; 0.98)
Child	0.79 (0.75; 0.84)	n/a	0.79 (0.75; 0.84)	0.77 (0.73; 0.81)	0.77 (0.73; 0.81)
Age at marriage	0.98 (0.97; 0.98)	n/a	0.94 (0.94; 0.94)	0.94 (0.94; 0.95)	0.94 (0.94; 0.95)
Externalizing			5.37 (5.17; 5.58)	5.04 (4.86; 5.24)	5.05 (4.86; 5.24)
Internalizing			5.68 (5.43; 5.94)	5.51 (5.26; 5.76)	5.50 (5.26; 5.76)
FGRS_SA_				1.23 (1.22; 1.25)	1.29 (1.27; 1.31)
FGRS_SA_×divorce					0.90 (0.88; 0.93)^b^
Males	Model A	Model A2	Model B	Model C	Model C2
Divorce	2.66 (2.55; 2.77)	n/a	1.70 (1.63; 1.78)	1.66 (1.59; 1.74)	1.72 (1.64; 1.80)
Year of birth	1.03 (1.03; 1.04)	n/a	1.01 (1.01; 1.01)	1.01 (1.01; 1.01)	1.01 (1.01; 1.01)
Parental education	0.93 (0.92; 0.94)	n/a	0.95 (0.95; 0.96)	0.96 (0.95; 0.96)	0.96 (0.95; 0.97)
Child	0.94 (0.89; 0.99)	n/a	0.95 (0.90; 1.00)	0.93 (0.88; 0.98)	0.93 (0.88; 0.98)
Age at marriage	1.00 (1.00; 1.01)	n/a	0.98 (0.98; 0.98)	0.98 (0.98; 0.99)	0.98 (0.98; 0.99)
Externalizing			3.22 (3.11; 3.34)	3.06 (2.94; 3.17)	3.04 (2.94; 3.17)
Internalizing			4.28 (4.09; 4.47)	4.14 (3.96; 4.34)	4.14 (3.96; 4.34)
FGRSsa				1.22 (1.21; 1.24)	1.27 (1.25; 1.29)
FGRS_SA_×divorce					0.92 (0.90; 0.94)^c^

Results are presented for the full sample, controlling for sex, followed by sex-stratified analyses.

FGRS_SA_ = family genetic risk score for suicide attempt. Results for model A2 are not presented for sex-stratified analyses as this model tested the effect of an interaction between sex and divorce.

a FGRS_SA_×divorce term is presented in the table on the multiplicative scale. To improve interpretability, we also estimated the relative excess risk due to interaction (RERI) and synergy index (SI). Values for the full sample were: RERI = 0.03 (0.00; 0.06); SI = 1.02 (1.00; 1.05).

b RERI = 0.02 (−0.03; 0.06); SI = 1.01 (0.98; 1.05).

c RERI = 0.02 (−0.03; 0.06); SI = 1.02 (0.97; 1.07).
