# Supplementary material for: Divorce and risk of suicide attempt: a Swedish national study
Source: Psychol Med. Author manuscript; Available in PMC 2025 Jun 1. (PMC11551852; doi:10.1017/S0033291723003513)
Supplement: Supplementary Material [file NIHMS2031893-supplement-Supplementary_Material.docx]

**Supplementary Material**

**Divorce and risk of suicide attempt: A Swedish national study**

Alexis C. Edwards, PhD, Henrik Ohlsson, PhD, Jessica E. Salvatore, PhD, Mallory E. Stephenson, MS, Casey Crump, MD, PhD, Jan Sundquist, MD, PhD, Kristina Sundquist, MD, PhD,^*^ Kenneth S. Kendler, MD^*^

*Joint senior authors

**Supplementary Methods**

*Registry descriptions*

Multi-Generation Register. See: <https://www.scb.se/en/finding-statistics/statistics-by-subject-area/other/other/other-publications-non-statistical/pong/publications/multi-generation-register-2016/>

Register of the total population. See: <https://www.scb.se/contentassets/8f66bcf5abc34d0b98afa4fcbfc0e060/rtb-bar-2016-eng.pdf>

National Patient Register: <https://www.socialstyrelsen.se/en/statistics-and-data/registers/national-patient-register/>

The Swedish Twin Register: <https://ki.se/en/research/the-swedish-twin-registry>

Primary Care data: See Sundquist, Ohlsson, Sundquist, and Kendler (2017).

*Variable descriptions*

Supplementary Table 1 includes descriptions of all variables used in the current study. Note that Statistics Sweden does not collect data on race/ethnicity.

*Family genetic risk score derivation*

Supplementary Table 2 details the information used and process applied in the derivation of family genetic risk scores (FGRS).

*Difference in difference models*

The underlying assumption in the difference-in-difference model is that the trend in suicide attempt (SA) among cases (those who divorced) would have been the same as the trend in SA among matched control subjects in the absence of a divorce. To validate this assumption, we included history of SA registrations among cases and controls for 6 years before the divorce. A causal interpretation is supported by a similar trend in the risk for SA before the divorce, but a different trend after the divorce. We used a three-level linear probability model with individual SA (yes or no) during the 12 specified time periods nested within individuals that in turn are nested within each stratum with case and their control. In the model, we included a dummy variable defining case or control and dummy variables for the different periods, except within 5-6 years before divorce that was used as a reference. Finally, we included interaction terms between the 11 time dummy variables on the one hand and the case or control covariate on the other. A significant interaction term suggests that the slope of SA was steeper among divorced than among their matched controls during. The use of this linear probability model is warranted as an alternative logit model would not give us the ability to explore the common trend assumption.

*Cox proportional hazards models*

In the Cox regression models we investigated several features. First, we tested it the effect of divorce was attenuated with time. This was done by including 3 additional terms (in Model C) that started 1,2 and 5 years after the divorce. Significant estimates, and HRs below 1, suggests that the effect of divorce on SA is attenuated with time. Second, we tested it the effect of divorce was attenuated based on length of marriage. This was done by including 5 terms (in Model C) that estimated the effect of divorce at different times since marriage. For simplicity we divided the follow-up time into quintiles and thereby divided the mean effect of divorce into 5 bins (0-2.6 years; 2.7-6.7 years; 6.8-13.4 years; 13.5-22.5 years; 22.6+ years). Third, in a secondary analysis, based on results from the difference-in-difference analysis, we subtracted 2 years from the date of actual divorce and included this new variable in the models. As we treat the variable as time dependent, it suggests that the individual is “exposed” two years prior to the actual date of divorce. Finally, to investigate the possibility of reverse causality (i.e., SA causes divorce), we reversed divorce and SA in the models so that SA was treated as a time dependent covariate while time to divorce was the outcome variable. For these four approaches we also used the co-relative design as explained below.

*Co-relative models*

Co-relative models are an extension of the co-twin control model (McGue, Osler, & Christensen, 2010). This approach facilitates causal inference by accounting for potential familial confounders (genes and family environment) that jointly contribute to risk for both a putative risk factor (here, divorce) and the outcome of interest. Monozygotic (MZ) twins share 100% of their genes and a large part of environmental factors suggesting that the HR for MZ twins is controlled for all possible confounding by genes and shared environment. Full- and half-siblings and cousins share, respectively, on average 50%, 25% and 12.5% of their genes identical by descent. Thus, a greater degree of genetic confounding is accounted for among relative pairs with higher genetic correlations. We combined all four samples (i.e., twin, full-, half-siblings, and cousins) into one dataset in which we performed two analyses. The first allowed all parameters for each sample to be independent (i.e., similar to four separate analyses); this analysis is referred to as the “Observed” model. In the second, we modeled the association between divorce and SA with two parameters: one main effect and one as a linear function of the genetic resemblance; this analysis is referred as the “Predicted” model. The HR for the second parameter gives an indication of the size of the familial confounding. If the second model fitted the data well, as indexed by the Akaike’s Information Criteria (AIC), we also obtained an improved estimation of the association among all relatives, but especially MZ twins, where the data was sparse.

**Supplementary Table 1.** Description of all variables included in the current study.

| **Variables** | **Registers Used** | **Definition** |
| --- | --- | --- |
| Suicide Attempt (SA) | National Patient Register and Primary Care Registry | ICD10: X60-X84 and Y10-Y34  ICD9: E950-E959 and E980-E989 |
| Divorce | Register of the total population | Date of registration of divorce |
| Parental Education | Register of the total population | Education was measured on a seven-point ordered categorical scale. We translated it to 7 years, 9 years, 11 year, 12 years, 14 years, 17, years, or 20 years, and took the mean across parents. |
| Child | Multi-Generation Register | Date of birth of first child. This variable was included to account for both de facto and de jure differences in rates of divorce based on whether the married couple had a child. In Sweden, parents seeking a divorce must undergo a formal waiting period, unlike married couples without children. |
| Externalizing | National Patient Register, Primary Care Registry, Crime Register, and Suspicion Register | Alcohol Use Disorder (AUD) was identified in the Swedish medical and mortality registries by ICD codes: ICD9: V79B, 305A, 357F, 571A-D, 425F, 535D, 291, 303, 980; ICD 10: E244, G312, G621, G721, I426, K292, K70, K852, K860, O354, T51, F10); in the Swedish Criminal Register and the Swedish Suspicion Register with at least two registrations of drunk driving (suspicion code 3005, law 1951:649 (paragraph 4 and 4A)) or drunk in charge of a maritime vessel (suspicion code 3201, law 1994:1009 (chapter 20, paragraph 4 and 5)); in the Prescribed Drug Register by the drugs disulfiram (Anatomical Therapeutic Chemical (ATC) Classification System N07BB01), acamprosate (N07BB03), and naltrexone (N07BB04).  Drug Use Disorder (DUD) was identified in the Swedish medical and mortality registries by ICD codes (ICD8: Drug dependence (304); ICD9: Drug psychoses (292) and Drug dependence (304); ICD10: Mental and behavioral disorders due to psychoactive substance use (F10-F19), except those due to alcohol (F10) or tobacco (F17)); in the Suspicion Register by codes 3070, 5010, 5011, and 5012, that reflect crimes related to DA; and in the Crime Register by references to laws covering narcotics (law 1968:64, paragraph 1, point 6) and drug-related driving offences (law 1951:649, paragraph 4, subsection 2 and paragraph 4A, subsection 2). DA was identified in individuals (excluding those suffering from cancer) in the Prescribed Drug Register who had retrieved (in average) more than four defined daily doses a day for 12 months from either of Hypnotics and Sedatives (Anatomical Therapeutic Chemical (ATC) Classification System N05C and N05BA).  Criminal behavior (CB) was identified by registration in the Swedish Crime (or conviction) register which excluded convictions for minor crimes like traffic infractions. or Opioids (ATC: N02A).  Externalizing behavior was a combination of AUD, DUD and CB. |
| Internalizing | National Patient Register and Primary Care Registry | ICD-8: 296.2, 298.0, 300.4; ICD-9: 296.2, 296.4, 298.0, 300.4; ICD-10: F32, F33. AND  ICD-8: 300.0, 300.2; ICD-9: 300A, 300C; ICD-10: F40, F41 |

**Supplementary Table 2.** Description of family genetic risk score (FGRS) derivation.

| The dataset for the calculations includes:  Column1 = Identification number of the proband (Born 1932-1995)  Column2 = Identification number of the relative (1st to 5th degree relatives)  Column3 = Proportion of shared additive genetic effects (0.03125 to 0.50) with the proband  Column4 = Year of Birth of relative  Column5 = Sex of relative  Column6 = Age at registration for trait  Column7 = Age at end of follow-up (2017-12-31 or age at death, or age at emigration whichever came first) |
| --- |
| **Step 1:** Using all unique relatives with a registration for the disorder, we non-parametrically estimated the distribution of *Age at first registration*. The empirical distribution is used to obtain weights for relatives without a registration for the disorder, in order to account for the proportion of the time-at-risk period they had completed at the end of follow-up. For example, for relatives at age x at end of follow-up, the weight corresponds to the proportion of relatives registered for the trait that had been registration at age x. For relatives born prior to 1958 we subtracted age at the end of follow-up with the following formula: 1958 - Year of birth of relative. This modification was done in order to control for registration effects (i.e., most registers in Sweden start in 1973 suggesting that relatives from early birth cohorts do not have the possibility to be registered at younger ages). Note that all relatives with the disorder are weighted one. |
| **Step 2:** Transform the binary variable (trait yes/no) into a z-score based on the threshold for each trait. The underlying liability of the individual is not assessable. Instead, we estimated the mean of the underlying liability to obtain sex and birth decade specific Z-scores for relatives with the trait registration and relatives without the trait. We generate n random numbers from a N (0, 1) distribution and estimate the mean for relatives registered with the disorder (i.e., mean of the observations above the threshold) and for relatives without a registration (i.e., mean of all observation below the threshold). The thresholds are calculated for each decade of birth and sex. |
| **Step 3**: Correct for cohabitation effects. To estimate the cohabitation effect (i.e., “shared environment”), we created a database with all individuals in the Swedish population born in Sweden 1955-1990. We also included the number of years, during ages 0-15, that individuals resided in the same household as their biological father. We thereby were able to define two kinds of families: i) “not-lived-with” father families (offspring never resided for more than 1 year in the same household or in the same community as their biological father); ii) “lived-with” father (offspring resided a minimum of 13 year in the same household as their biological father. We performed a logistic regression model with the binary trait in offspring as outcome and the binary trait in father, type of father, and their interaction as predictors. We used the interaction term as the difference of effect between genes only and genes + environment. The same approach was performed for half-siblings where we compared those who were reared together versus reared apart. The following interaction terms were used in the calculations for each of our 7 main disorders:   \|  \| Parent/Children \| Siblings \| \| --- \| --- \| --- \| \| SA \| .66 \| .76 \| |
| **Step 4:** Calculate the product for each relative using the four components:   1. Z-score (reflecting sex and year of birth adjusted rates) 2. Weight (reflecting the proportion of risk period they had completed) 3. Cohabitation effects 4. Proportion of shared genetic effects (0.03125 – 0.5) with the proband |
| **Step 5:** Average the product calculated in step 4 across all relatives to a proband |
| **Step 6**: Correct for the number of relatives. We multiplied the results from step 5 with a shrinkage factor. Shrinkage factor (SF): B/(B+A/C). It produces more shrinkage if B and C are small and A is large.   1. the variance of the z-score of the disorder across all relatives, 2. the variance in the mean z-score across all probands, 3. the weighted number of relatives for each proband (sum of Column 3 across each proband). |
| **Step 7:** Correct for difference by year of birth and county differences. There are 21 counties in Sweden. For each proband we used the county they had resided in during the maximum number of years (measured from 1969 and onwards) We standardized the risk score by year of birth and county of the proband into a z-score with mean 0 and SD 1. This was then used as the FGRS in the analyses. |

**Supplementary Table 3.** Hazard ratios and 95% confidence intervals from co-relative models estimating the association between divorce and suicide attempt. Results are presented from models including the sexes combined as well as for sex-stratified models. As described in the Supplementary Methods section, we calculated estimates based on observed data (“Observed”) and for predicted data (“Predicted”) based on genetic relatedness between members of each pair type, and compared model fit using Akaike’s Information Criterion (AIC). AIC values are presented for each model. The top panel reflects results from Model A from the main text, which is adjusted for sociodemographic covariates. The bottom panel reflects results from Model B, which is further adjusted for externalizing and internalizing registrations.

|  | **Sexes Combined** | | **Females** | | **Males** | |
| --- | --- | --- | --- | --- | --- | --- |
| Model A^1^ | Observed | Predicted | Observed | Predicted | Observed | Predicted |
| Cousins | 2.77 (2.65; 2.88) | 2.79 (2.68; 2.90) | 3.09 (2.86; 3.34) | 2.99 (2.78; 3.21) | 2.53 (2.32; 2.77) | 2.53 (2.32; 2.75) |
| Half Siblings | 2.78 (2.56, 3.02) | 2.65 (2.57; 2.73) | 2.30 (2.01; 2.65) | 2.76 (2.61; 2.92) | 2.50 (2.08; 3.01) | 2.49 (2.33; 2.66) |
| Full Siblings | 2.38 (2.26; 2.50) | 2.40 (2.28; 2.52) | 2.41 (2.19; 2.64) | 2.35 (2.15, 2.57) | 2.39 (2.14, 2.66) | 2.41 (2.17; 2.68) |
| Twins | 2.92 (1.05; 8.14) | 1.97 (1.73; 2.24) | 2.03 (0.57, 7.21) | 1.70 (1.35; 2.15) | 4.85 (0.79; 29.7) | 2.27 (1.72; 2.99) |
| AIC | 51217.237 | 51214.493 | 14747.031 | 14747.443 | 11271.208 | 11267.798 |
|  |  |  |  |  |  |  |
| Model B^2^ | Observed | Predicted | Observed | Predicted | Observed | Predicted |
| Cousins | 1.94 (1.85; 2.04) | 1.95 (1.87; 2.04) | 2.21 (2.01; 2.42) | 2.14 (1.96; 2.33) | 1.65 (1.50; 1.83) | 1.63 (1.49; 1.80) |
| Half Siblings | 2.00 (1.81; 2.20) | 1.91 (1.84; 1.98) | 1.77 (1.48; 2.10) | 2.02 (1.89; 2.16) | 1.62 (1.32; 1.99) | 1.70 (1.58; 1.83) |
| Full Siblings | 1.80 (1.70; 1.91) | 1.82 (1.72; 1.93) | 1.82 (1.63; 2.03) | 1.81 (1.63; 2.01) | 1.82 (1.61; 2.06) | 1.83 (1.62; 2.06) |
| Twins | 4.10 (1.38; 12.1) | 1.66 (1.43; 1.93) | 3.88 (1.21; 12.4) | 1.45 (1.10; 1.90) | 5.07 (0.54; 47.1) | 2.13 (1.56; 2.90) |
| AIC | 41883.369 | 41881.592 | 11412.763 | 11411.597 | 9577.921 | 9574.674 |

AIC=Akaike’s Information Criterion

^1^Model A is adjusted for year of birth, parental education, parenthood status, and age at marriage.

^2^Model B is adjusted for the variables in Model A as well as externalizing and internalizing registrations.

**Supplementary Table 4.** Hazard ratios and 95% confidence intervals for the association between divorce and suicide attempt as a function of time elapsed since divorce registration. Results are presented for the sexes combined and for sex-stratified analyses. Models are adjusted for sociodemographic covariates and registrations for externalizing and internalizing, as in Model C in the main text.

| Time since divorce registration | Sexes Combined | Females | Males |
| --- | --- | --- | --- |
| 0 to 1 years | 2.57 (2.43; 2.72) | 2.91 (2.69; 3.14) | 2.20 (2.01; 2.41) |
| 1 to 2 years | 2.21 (2.07; 2.36) | 2.55 (2.35; 2.77) | 1.83 (1.65; 2.02) |
| 2 to 5 years | 1.74 (1.66; 1.82) | 1.79 (1.68; 1.90) | 1.68 (1.57; 1.80) |
| More than 5 | 1.47 (1.42; 1.53) | 1.41 (1.34; 1.49) | 1.51 (1.43; 1.59) |

**Supplementary Table 5.** Hazard ratios and 95% confidence intervals for the association between divorce and suicide attempt as a function of marriage length. Results are presented for the sexes combined and for sex-stratified analyses. Models are adjusted for sociodemographic covariates and registrations for externalizing and internalizing, as in Model C in the main text.

| Length of marriage | Sexes Combined | Females | Males |
| --- | --- | --- | --- |
| 0 - 2.7 years (lowest decile) | 3.40 (2.98; 3.87) | 3.39 (2.85; 4.04) | 3.33 (2.73; 4.06) |
| 2.7 - 6.8 years (10-25%) | 2.37 (2.23; 2.52) | 2.61 (2.40; 2.83) | 2.12 (1.93; 2.32) |
| 6.8 - 13.5 years (25-50%) | 1.81 (1.73; 1.90) | 1.96 (1.83; 2.09) | 1.65 (1.54; 1.77) |
| 13.5 - 22.6 years (50-75%) | 1.44 (1.37; 1.51) | 1.39 (1.30; 1.48) | 1.47 (1.37; 1.58) |
| 22.6+ years (75%+) | 1.29 (1.20; 1.39) | 1.20 (1.09; 1.33) | 1.36 (1.20; 1.53) |

**Supplementary Table 6.** Hazard ratios and 95% confidence intervals from Cox regressions estimating the association between divorce and first suicide attempt, where the “divorce” exposure is set to two years prior to divorce registration. Results are presented for the full sample, controlling for sex, followed by sex-stratified analyses.

| Full sample | Model A | Model A2 | Model B | Model C | Model C2 |
| --- | --- | --- | --- | --- | --- |
| Divorce | 3.93 (3.82; 4.04) | 4.35 (4.20; 4.51) | 2.50 (2.43; 2.57) | 2.43 (2.36; 2.50) | 2.51 (2.43; 2.58) |
| Year of birth | 1.03 (1.03; 1.03) | 1.03 (1.03; 1.03) | 0.99 (0.99; 0.99) | 0.99 (0.99; 0.99) | 0.99 (0.99; 0.99) |
| Sex (M vs F) | 1.10 (1.08; 1.13) | 1.24 (1.20; 1.28) | 0.98 (0.95; 1.01) | 0.99 (0.96; 1.01) | 0.99 (0.96; 1.01) |
| Parental education | 0.94 (0.94; 0.95) | 0.94 (0.94; 0.95) | 0.96 (0.96; 0.97) | 0.97 (0.96; 0.97) | 0.97 (0.96; 0.97) |
| Child | 0.90 (0.87; 0.94) | 0.90 (0.87; 0.93) | 0.90 (0.86; 0.93) | 0.87 (0.84; 0.91) | 0.87 (0.84; 0.91) |
| Age at marriage | 0.99 (0.99; 1.00) | 0.99 (0.99; 1.00) | 0.96 (0.96; 0.97) | 0.97 (0.96; 0.97) | 0.97 (0.96; 0.97) |
| Sex * Divorce |  | 0.80 (0.76; 0.84) |  |  |  |
| Externalizing |  |  | 3.86 (3.76; 3.97) | 3.65 (3.55; 3.75) | 3.65 (3.55; 3.75) |
| Internalizing |  |  | 4.64 (4.50; 4.79) | 4.50 (4.37; 4.65) | 4.50 (4.36; 4.65) |
| FGRS_SA_ |  |  |  | 1.22 (1.21; 1.23) | 1.28 (1.27; 1.30) |
| FGRS_SA_ * Divorce |  |  |  |  | 0.92 (0.90; 0.93)^1^ |
|  |  |  |  |  |  |
| Females | Model A | Model A2 | Model B | Model C | Model C2 |
| Divorce | 4.31 (4.15; 4.48) | n/a | 2.63 (2.52; 2.74) | 2.55 (2.44; 2.66) | 2.65 (2.54; 2.76) |
| Year of birth | 1.02 (1.02; 1.02) | n/a | 0.97 (0.97; 0.97) | 0.97 (0.97; 0.97) | 0.97 (0.97; 0.97) |
| Parental education | 0.95 (0.94; 0.95) | n/a | 0.97 (0.96; 0.97) | 0.97 (0.97; 0.98) | 0.97 (0.97; 0.98) |
| Child | 0.84 (0.79; 0.88) | n/a | 0.83 (0.79; 0.87) | 0.80 (0.76; 0.85) | 0.80 (0.76; 0.85) |
| Age at marriage | 0.98 (0.98; 0.99) | n/a | 0.95 (0.94; 0.95) | 0.95 (0.95; 0.95) | 0.95 (0.95; 0.95) |
| Externalizing |  |  | 4.94 (4.76; 5.13) | 4.66 (4.48; 4.84) | 4.66 (4.49; 4.84) |
| Internalizing |  |  | 5.35 (5.11; 5.59) | 5.19 (4.96; 5.43) | 5.19 (4.96; 5.43) |
| FGRS_SA_ |  |  |  | 1.22 (1.21; 1.24) | 1.29 (1.27; 1.32) |
| FGRS_SA_ * Divorce |  |  |  |  | 0.91 (0.89; 0.93)^2^ |
|  |  |  |  |  |  |
| Males | Model A | Model A2 | Model B | Model C | Model C2 |
| Divorce | 3.51 (3.37; 3.66) | n/a | 2.34 (2.24; 2.44) | 2.29 (2.19; 2.38) | 2.35 (2.25; 2.45) |
| Year of birth | 1.03 (1.03; 1.04) | n/a | 1.01 (1.01; 1.01) | 1.01 (1.01; 1.01) | 1.01 (1.01; 1.01) |
| Parental education | 0.93 (0.93; 0.94) | n/a | 0.96 (0.95; 0.96) | 0.96 (0.95; 0.97) | 0.96 (0.95; 0.97) |
| Child | 0.98 (0.93; 1.04) | n/a | 0.98 (0.93; 1.04) | 0.97 (0.91; 1.02) | 0.97 (0.91; 1.02) |
| Age at marriage | 1.01 (1.00; 1.01) | n/a | 0.98 (0.98; 0.99) | 0.99 (0.98; 0.99) | 0.99 (0.98; 0.99) |
| Externalizing |  |  | 3.01 (2.90; 3.12) | 2.86 (2.76; 2.97) | 2.86 (2.76; 2.97) |
| Internalizing |  |  | 4.03 (3.86; 4.22) | 3.92 (3.74; 4.10) | 3.92 (3.74; 4.10) |
| FGRS_SA_ |  |  |  | 1.22 (1.20; 1.23) | 1.27 (1.25; 1.29) |
| FGRS_SA_ * Divorce |  |  |  |  | 0.93 (0.90; 0.95)^3^ |

FGRS_SA_=family genetic risk score for suicide attempt. Results for Model A2 are not presented for sex-stratified analyses as this model tested the effect of an interaction between sex and divorce.

^1^FGRS_SA_*divorce term is presented in the table on the multiplicative scale. To improve interpretability, we also estimated the relative excess risk due to interaction (RERI) and synergy index (SI). Values for the full sample were: RERI=0.16 (0.12; 0.20); SI=1.09 (1.07; 1.11)

^2^RERI=0.16 (0.11; 0.22); SI=1.08 (1.06; 1.11)

^3^RERI=0.14 (0.08; 0.20); SI=1.09 (1.05; 1.12)

**Supplementary Table 7.** Hazard ratios and 95% confidence intervals from co-relative models estimating the association between divorce and suicide attempt, where the “divorce” exposure includes the two years prior to divorce registration. Results are presented from models including the sexes combined as well as for sex-stratified models. As described in the Supplementary Methods section, we calculated estimates based on observed data (“Observed”) and for predicted data (“Predicted”) based on genetic relatedness between members of each pair type, and compared model fit using Akaike’s Information Criterion (AIC). AIC values are presented for each model. The top panel reflects results from Model A from the main text, which is adjusted for sociodemographic covariates. The bottom panel reflects results from Model B, which is further adjusted for externalizing and internalizing registrations.

|  | **Sexes Combined** | | **Females** | | **Males** | |
| --- | --- | --- | --- | --- | --- | --- |
| Model A^1^ | Observed | Predicted | Observed | Predicted | Observed | Predicted |
| Cousins | 3.95 (3.79; 4.12) | 4.00 (3.85; 4.17) | 4.37 (4.04; 4.73) | 4.32 (4.01; 4.65) | 3.54 (3.25; 3.87) | 3.62 (3.33; 3.94) |
| Half Siblings | 4.15 (3.82; 4.52) | 3.79 (3.68; 3.91) | 3.68 (3.17; 4.26) | 4.01 (3.78; 4.24) | 4.19 (3.46; 5.08) | 3.52 (3.29; 3.75) |
| Full Siblings | 3.35 (3.19; 3.53) | 3.39 (3.23; 3.57) | 3.51 (3.19; 3.87) | 3.44 (3.13; 3.77) | 3.21 (2.88; 3.58) | 3.31 (2.98; 3.68) |
| Twins | 2.71 (1.12; 6.56) | 2.72 (2.38; 3.10) | 1.29 (0.44; 3.72) | 2.53 (1.99; 3.23) | 6.97 (1.09; 44.7) | 2.94 (2.24; 3.90) |
| AIC | 47932.168 | 47931.788 | 13724.555 | 13722.341 | 10630.048 | 10629.509 |
|  |  |  |  |  |  |  |
| Model B^2^ | Observed | Predicted | Observed | Predicted | Observed | Predicted |
| Cousins | 2.73 (2.61; 2.85) | 2.77 (2.66; 2.90) | 3.14 (2.88; 3.42) | 3.13 (2.88; 3.39) | 2.30 (2.10; 2.52) | 2.35 (2.15, 2.56) |
| Half Siblings | 3.03 (2.77; 3.32) | 2.70 (2.61; 2.79) | 2.90 (2.46; 3.43) | 2.94 (2.76; 3.13) | 2.83 (2.32; 3.46) | 2.39 (2.23, 2.56) |
| Full Siblings | 2.51 (2.37; 2.65) | 2.56 (2.42; 2.70) | 2.59 (2.33; 2.88) | 2.59 (2.34; 2.86) | 2.39 (2.13; 2.68) | 2.47 (2.21; 2.76) |
| Twins | 3.65 (1.52; 8.77) | 2.29 (1.99; 2.64) | 2.33 (0.80; 6.79) | 2.01 (1.54; 2.62) | 6.97 (0.87; 56.1) | 2.64 (1.97; 3.53) |
| AIC | 40315.785 | 40317.141 | 10902.176 | 10898.218 | 9296.485 | 9295.510 |

AIC=Akaike’s Information Criterion

^1^Model A is adjusted for year of birth, parental education, parenthood status, and age at marriage.

^2^Model B is adjusted for the variables in Model A as well as externalizing and internalizing registrations.

**Supplementary Table 8.** Hazard ratios and 95% confidence intervals for the association between divorce and suicide attempt as a function of time elapsed since divorce registration. Here, the “divorce” exposure includes the two years prior to divorce registration; thus, the first two time bins precede formal marital dissolution. Results are presented for the sexes combined and for sex-stratified analyses. Models are adjusted for sociodemographic covariates and registrations for externalizing and internalizing, as in Model C in the main text.

| Time since divorce registration | Sexes Combined | Females | Males |
| --- | --- | --- | --- |
| 0 to 1 years | 2.98 (2.81; 3.17) | 3.19 (2.94; 3.46) | 2.78 (2.54; 3.05) |
| 1 to 2 years | 5.02 (4.79; 5.27) | 5.36 (5.02; 5.73) | 4.69 (4.37; 5.03) |
| 2 to 5 years | 2.62 (2.51; 2.73) | 3.00 (2.84; 3.17) | 2.22 (2.09; 2.36) |
| More than 5 | 1.80 (1.74; 1.87) | 1.79 (1.70; 1.87) | 1.79 (1.70; 1.89) |

**Supplementary Table 9.** Hazard ratios and 95% confidence intervals for the association between divorce and suicide attempt as a function of marriage length. Here, the “divorce” exposure includes the two years prior to divorce registration. Results are presented for the sexes combined and for sex-stratified analyses. Models are adjusted for sociodemographic covariates and registrations for externalizing and internalizing, as in Model C in the main text.

| Length of marriage | Sexes Combined | Females | Males |
| --- | --- | --- | --- |
| 0 - 2.7 years (lowest decile) | 4.89 (4.54; 5.26) | 5.28 (4.78; 5.84) | 4.41 (3.98; 4.96) |
| 2.7 - 6.8 years (10-25%) | 3.32 (3.14; 3.51) | 3.62 (3.35; 3.92) | 3.04 (2.81; 3.29) |
| 6.8 - 13.5 years (25-50%) | 2.36 (2.25; 2.47) | 2.64 (2.47; 2.82) | 2.10 (1.96; 2.24) |
| 13.5 - 22.6 years (50-75%) | 1.81 81.72; 1.90) | 1.78 (1.67; 1.91) | 1.80 (1.67; 1.94) |
| 22.6+ years (75%+) | 1.45 (1.34; 1.56) | 1.35 (1.22; 1.50) | 1.52 (1.35; 1.72) |

**Supplementary Table 10.** Hazard ratios and 95% confidence intervals from Cox regressions estimating the association between suicide attempt and divorce in full sample (sexes combined).

|  | Model A | Model B |
| --- | --- | --- |
| Suicide attempt | 2.24 (2.21; 2.28) | 1.54 (1.51; 1.57) |
| Year of birth | 1.00 (1.00; 1.00) | 0.99 (0.99; 0.99) |
| Sex (M vs F) | 1.03 (1.03; 1.04) | 0.99 (0.98; 0.99) |
| Parental education | 0.90 (0.90; 0.90) | 0.93 (0.92; 0.93) |
| Child | 0.52 (0.51; 0.52) | 0.53 (0.52; 0.53) |
| Age at marriage | 0.96 (0.96; 0.96) | 0.95 (0.95; 0.95) |
| Externalizing registration |  | 2.03 (2.01; 2.05) |
| Internalizing registration |  | 2.02 (2.00; 2.04) |

**Supplementary Table 11.** Hazard ratios and 95% confidence intervals from co-relative models estimating the association between suicide attempt and later divorce. Results are presented from models including the sexes combined. As described in the Supplementary Methods section, we calculated estimates based on observed data (“Observed”) and for predicted data (“Predicted”) based on genetic relatedness between members of each pair type, and compared model fit using Akaike’s Information Criterion (AIC). AIC values are presented for each model.

|  | **Sexes Combined** | |
| --- | --- | --- |
| Model A^1^ | Observed | Predicted |
| Cousins | 2.12 (1.08; 2.17) | 2.10 (2.05; 2.14) |
| Half Siblings | 1.89 (1.82; 1.97) | 2.02 (1.98; 2.05) |
| Full Siblings | 1.89 (1.84; 1.95) | 1.87 (1.82; 1.92) |
| Twins | 1.72 (1.06; 2.78) | 1.60 (1.49; 1.73) |
| AIC | 782787.64 | 782790.47 |
|  |  |  |
| Model B^2^ | Observed | Predicted |
| Cousins | 1.58 (1.55; 1.62) | 1.56 (1.53; 1.59) |
| Half Siblings | 1.42 (1.36; 1.47) | 1.53 (1.50; 1.55) |
| Full Siblings | 1.48 (1.44; 1.53) | 1.46 (1.42; 1.50) |
| Twins | 1.15 (0.69; 1.95) | 1.34 (1.24; 1.44) |
| AIC | 759921.45 | 759926.80 |

AIC=Akaike’s Information Criterion

^1^Model A is adjusted for year of birth, parental education, parenthood status, sex, and age at marriage.

^2^Model B is adjusted for the variables in Model A as well as externalizing and internalizing registrations.

**Supplementary Table 12.** Hazard ratios and 95% confidence intervals from exploratory analyses including spousal registrations for psychopathology and suicide attempt. Spousal registrations were included as time-dependent covariates. This model complements Model C from Table 2 in the primary text.

| Full sample | Model C (primary text) | Model C Spouse |
| --- | --- | --- |
| Divorce | 1.73 (1.68; 1.79) | 1.64 (1.59; 1.70) |
| Year of birth | 0.99 (0.99; 0.99) | 0.99 (0.99; 0.99) |
| Sex (M vs F) | 0.98 (0.96; 1.01) | 1.03 (1.00; 1.06) |
| Parental education | 0.97 (0.96; 0.97) | 0.97 (0.96; 0.97) |
| Child | 0.84 (0.81; 0.87) | 0.85 (0.82; 0.89) |
| Age at marriage | 0.96 (0.96; 0.97) | 0.96 (0.96; 0.97) |
| Externalizing | 3.93 (3.82; 4.04) | 3.72 (3.61; 3.83) |
| Internalizing | 4.77 (4.62; 4.99) | 4.74 (4.59; 4.90) |
| FGRS_SA_ | 1.23 (1.22; 1.24) | 1.23 (1.22; 1.24) |
| SA_Spouse_ |  | 1.56 (1.48; 1.65) |
| Externalizing_Spouse_ |  | 1.21 (1.17; 1.25) |
| Internalizing_Spouse_ |  | 0.97 (0.94; 1.01) |
|  |  |  |
| Females | Model C (primary text) | Model C Spouse |
| Divorce | 1.77 (1.69; 1.84) | 1.70 (1.63; 1.78) |
| Year of birth | 0.97 (0.97; 0.97) | 0.97 (0.97; 0.97) |
| Parental education | 0.97 (0.97; 0.98) | 0.97 (0.97; 0.98) |
| Child | 0.77 (0.73; 0.81) | 0.76 (0.72; 0.81) |
| Age at marriage | 0.94 (0.94; 0.95) | 0.94 (0.94; 0.95) |
| Externalizing | 5.04 (4.86; 5.24) | 4.87 (4.68; 5.08) |
| Internalizing | 5.51 (5.26; 5.76) | 5.55 (5.30; 5.83) |
| FGRS_SA_ | 1.23 (1.22; 1.25) | 1.23 (1.21; 1.25) |
| SA_Spouse_ |  | 1.61 (1.49; 1.75) |
| Externalizing_Spouse_ |  | 1.14 (1.09; 1.19) |
| Internalizing_Spouse_ |  | 0.87 (0.82; 0.93) |
|  |  |  |
|  |  |  |
| Males | Model C (primary text) | Model C Spouse |
| Divorce | 1.66 (1.59; 1.74) | 1.56 (1.49; 1.63) |
| Year of birth | 1.01 (1.01; 1.01) | 1.01 (1.00; 1.01) |
| Parental education | 0.96 (0.95; 0.96) | 0.96 (0.95; 0.97) |
| Child | 0.93 (0.88; 0.98) | 0.96 (0.91; 1.02) |
| Age at marriage | 0.98 (0.98; 0.99) | 0.98 (0.98; 0.99) |
| Externalizing | 3.06 (2.94; 3.17) | 2.90 (2.79; 3.01) |
| Internalizing | 4.14 (3.96; 4.34) | 4.07 (3.88; 4.27) |
| FGRS_SA_ | 1.73 (1.68; 1.79) | 1.22 (1.21; 1.24) |
| SA_Spouse_ |  | 1.53 (1.42; 1.65) |
| Externalizing_Spouse_ |  | 1.25 (1.19; 1.33) |
| Internalizing_Spouse_ |  | 1.03 (0.98; 1.08) |
|  |  |  |

**Supplementary Figure.** Difference-in-difference models

Cases (i.e., individuals that are divorced) are matched to three controls based on year of birth, sex, age at marriage (+/-1 years), FGRS_SA_ (quartiles of the distribution based on k-means clustering), and parental status; controls were not divorced at the time of the case’s divorce. The divorce had to occur prior to 2012-12-13 to allow for at least 6 years of follow up. The annual prevalence of suicide attempt is plotted beginning 5-6 years prior to the year of the cases’ divorce and ending 5-6 years later. The vertical black line represents the point of divorce. Estimates are provided for the sexes combined and stratified by sex.

**References**

McGue, Matt, Osler, Merete, & Christensen, Kaare. (2010). Causal inference and observational research: The utility of twins. *Perspectives on psychological science, 5*(5), 546-556.

Sundquist, J., Ohlsson, H., Sundquist, K., & Kendler, K. S. (2017). Common adult psychiatric disorders in Swedish primary care where most mental health patients are treated. *BMC Psychiatry, 17*(1), 235. doi:10.1186/s12888-017-1381-4
